# Anabolic-androgenic steroid testing as a tool for consumer engagement and harm reduction: a sequential explanatory mixed-method study

**DOI:** 10.1186/s12954-025-01270-4

**Published:** 2025-07-04

**Authors:** Timothy Piatkowski, Ross Coomber, Cameron Francis, Emma Kill, Geoff Davey, Sarah Cresswell, Alan White, Madeline Harding, Karen Blakey, Steph Reeve, Brooke Walters, Cheneal Puljević, Jason Ferris, Monica J. Barratt

**Affiliations:** 1 School of Applied Psychology, Griffith University, Mount Gravatt Campus, Gold Coast, QLD 4122 Australia; 2 Griffith Centre for Mental Health, Griffith University, Brisbane, Australia; 3 Queensland Injectors Voice for Advocacy and Action, Sunshine Coast, QLD Australia; 4 The Loop Australia, Brisbane, Australia; 5 Department of Sociology, Social Policy and Criminology, Faculty of Humanities and Social Sciences, University of Liverpool, Liverpool, UK; 6 Queensland Injectors Health Network, Brisbane, Australia; 7 School of Environment and Science, Griffith University, Brisbane, Australia; 8 School of Public Health, The University of Queensland, Brisbane, QLD Australia; 9 Centre for Health Services Research, The University of Queensland, Brisbane, QLD Australia; 10 Criminology and Justice Studies Social Equity Research Centre and Digital Ethnography Research Centre, RMIT University, Melbourne, Vic Australia; 11 National Drug and Alcohol Research Centre, UNSW Sydney, sydney, NSW Australia

**Keywords:** Anabolic-androgenic steroids, Image and performance enhancing drugs, Drug checking, Steroid checking, Harm reduction

## Abstract

**Introduction:**

Anabolic-androgenic steroid (AAS) use is widespread, yet regulation remains limited, exposing consumers to misidentified and contaminated products. This study expands AAS testing by enhancing purity analysis, identifying branding inconsistencies, and examining consumer responses. It aims to evaluate the impact of enhanced testing and feedback on consumer behaviour and harm reduction.

**Methods:**

This is a sequential explanatory mixed-methods study incorporating chemical analysis of community-submitted AAS samples and interviews with participants. Interviews used semi-structured formats, focusing on participants’ understanding of the testing results, and how it influenced their AAS usage decisions. We sampled from community harm reduction organisations across two drug checking sites in Queensland, Australia.

**Results:**

Between April 19 and August 16, 2024, 58 AAS samples were submitted. Chemical analysis was conducted using Radian-ASAP direct mass spectrometry and Orbitrap Liquid Chromatography Mass Spectrometry to verify the identity and dosage of the submitted AAS. Of the 46 analysable samples, 9 exhibited presence issues (i.e., the compound differed from expectations), while 15 demonstrated purity issues (i.e., the concentration was either too low or too high). Twenty-five AAS consumers were interviewed, with follow-up interviews conducted with 15 participants to assess their behaviour changes. Interviews (*N* = 40) indicated that consumers’ trust in the substances they used was reduced, leading to more cautious approaches and reconsideration of usage practices.

**Conclusion:**

This study demonstrates that chemical analysis can be a powerful tool in influencing AAS consumers’ practices, highlighting the need for further research on how testing, coupled with harm reduction interventions, can improve consumer safety and decision-making.

**Supplementary Information:**

The online version contains supplementary material available at 10.1186/s12954-025-01270-4.

## Introduction

Anabolic-androgenic steroids (AAS), synthetic derivatives of testosterone, are widely used by people seeking to enhance physical performance, wellbeing, and aesthetics [1]. Lifetime prevalence is estimated at 6.4% among men [2] and 4% among women [3]. Despite their widespread use, AAS remain largely unregulated, with over 66% of products containing undeclared, misidentified, or contaminated substances that pose serious health risks [4, 5]. Consumers of these unregulated products face potential harms such as cardiovascular disease, endocrine disruption, hepatotoxicity, and injection-related infections [6, 7]. Additionally, while psychological effects such as aggression, anxiety, dependence, and post-cycle depression are well-documented [8–11] these experiences are often overlooked in healthcare settings.

Harm reduction strategies have been successfully implemented for substances like opioids (e.g., supervised injecting rooms, needle exchange programs) [12, 13] and alcohol (e.g., safe drinking guidelines, support services) [14], but AAS-specific harm reduction interventions remain gravely underdeveloped [15, 16]. While drug checking programs provide chemical analysis of illicit substances [14], AAS testing has not been integrated into these models. This gap leaves consumers without reliable information about the products they are using, increasing the likelihood of harm [17, 18]. Furthermore, people who use AAS often report barriers to healthcare, including stigma, misinformation, and a lack of tailored services [19–24]. These conditions create a policy vacuum in which people are both criminalised and unsupported, pointing to an urgent need for integrated, health-based responses.

Preliminary research has sought to address this by implementing an AAS testing trial in Queensland, Australia [25] offering an early model for integrated harm reduction responses. The first ‘Wave’ of the trial provided chemical analysis of AAS samples and disseminated grouped results to consumers, revealing critical issues in product composition. However, this first Wave had limitations in fully characterising variability in purity, reporting product branding, and determining implications on consumer behaviours. This is especially critical, as consumers had previously expressed concerns that the limitations of the first Wave hindered their ability to make informed, effective changes to their AAS consumption. Considering these insights, the aim of this research is to critically evaluate the influence of additional data (‘Wave 2’) on consumer behaviour, assessing how more accurate, longitudinal feedback can empower consumers to engage in safer and more well-informed practices.

## Methods

### Study design and ethics

**Fig. 1:**
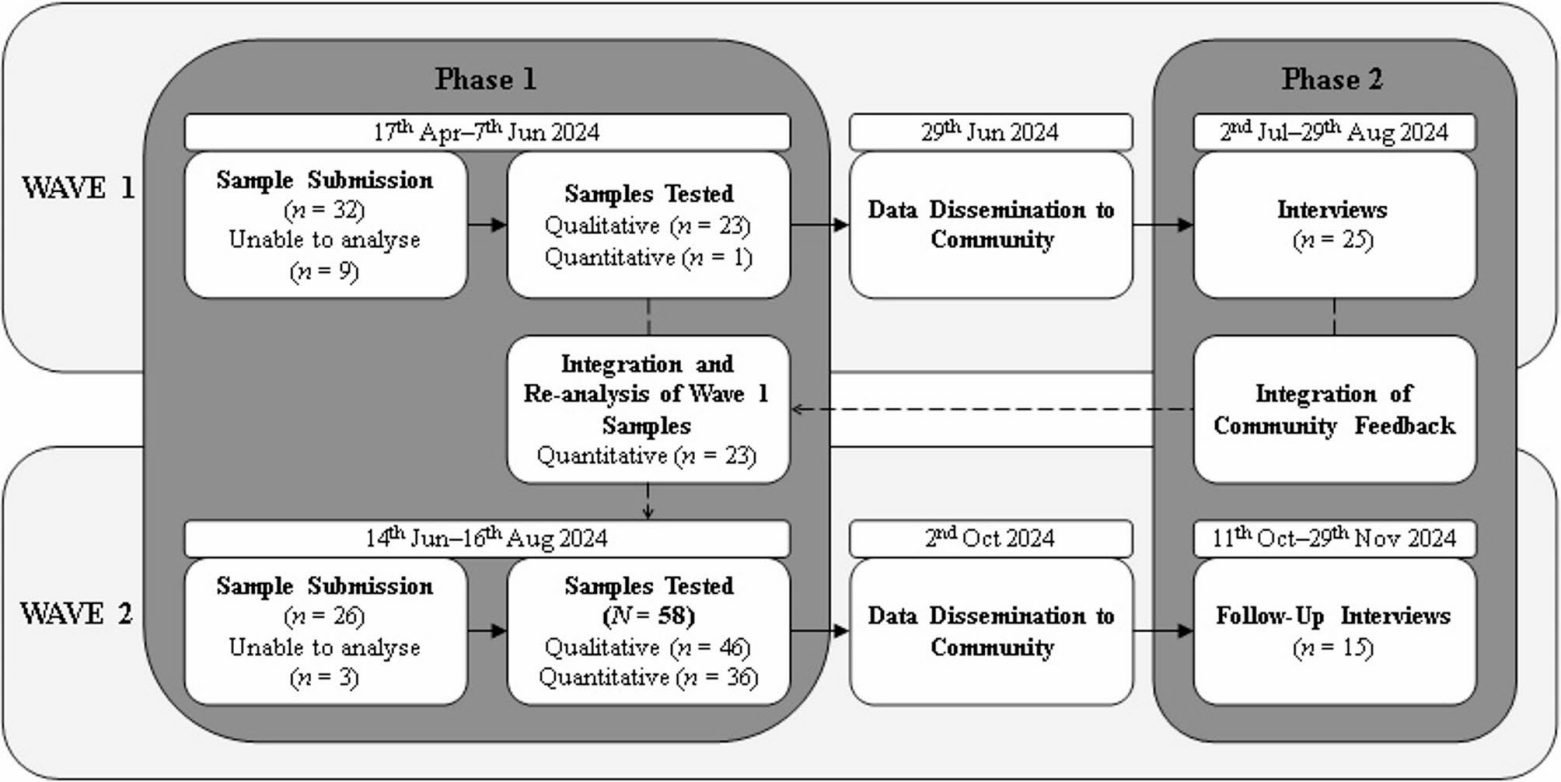
Sequential Explanatory Mixed-Methods Design and Timeline

This study used a sequential explanatory mixed-methods design [26]. The second Wave (‘Wave 2’) of the trial expanded the scope through enhanced purity precision, the identification of branding inconsistencies, and an increased sample size across multiple sites, and also facilitated follow-up with participants from the first Wave. This sequential design allowed for an in-depth evaluation of how the revised reporting influenced consumer behaviour. Ethical approval was granted by the Griffith University Human Research Ethics Committee (Approval: 2023/784). The study followed the COREQ [27] reporting guidelines (see supplemental materials). Figure 1 illustrates the phases of data collection, including sample submission, laboratory analysis, and subsequent time frames for result dissemination.

### Phase 1: chemical analysis of AAS samples

Participants were encouraged to submit AAS samples—including used vials with residual product, unopened vials, and oral tablets—to CheQpoint drug checking services in Brisbane and a second site on the Gold Coast, Queensland. Upon submission, all samples were logged, labelled, and were processed and securely stored at CheQpoint. Samples were then transported from CheQpoint sites to the Griffith University chemistry laboratory. Initial screening was performed using Radian-ASAP or FTIR (for tablets, capsules, and powders) to detect the presence of active compounds. When compounds of interest were identified, samples were re-extracted and analysed using LC-MS (Orbitrap 360) to confirm the identity and quantify the concentration. The resulting spectral data were cross-referenced against National Institute of Standards and Technology (NIST) libraries and available reference materials [4]. All results underwent multiple levels of data checking, including internal review within the chemistry team. Positive findings, especially those indicating mislabelled substances or unexpected pharmacological content, required formal sign-off by a senior chemist. These methods were previously validated for use with AAS in our prior work [24, 25].

Due to the complexity of these analytical methods, collection of samples, reference material purchasing, and batch processing, participants typically waited between 2 and 3 months between ‘Waves’ of data, a delay that differs significantly from on-site drug checking services offering near-immediate feedback [14]. These results were compiled and disseminated through posters at the collection site, as well as digital networks facilitated by community partners (see Table 1). Comprehensive findings from the analysis (collected between 19th April–16th August 2024) were made available via social media and community platforms.

**Table 1:** Testing summary

Expected Compound/s	Expected Concentration	Brand	As Expected?	Detected Compound/s	Concentration as Expected?	Detected Concentration
Wave 1: 19 APRIL 2024–7 JUNE 2024						
Trestolone − 7α-methyl-19-nortestosterone (MENT)	50 mg/mL	Apex Anabolics	No	Trestolone Acetate	N/A	-
Testosterone Propionate	100 mg/mL	Platinum	Yes	Testosterone Propionate	Yes	96 mg/mL (± 5% error = 91–101)
Nandrolone Phenylpropionate (NPP)	100 mg/mL	Platinum	Yes	Nandrolone Phenylpropionate	No - underdosed	87 mg/mL (± 5% error = 83–91)
Stanozolol (Winstrol)	10 mg	Sparta	Yes	Stanozolol	No - underdosed	3.3 mg
Testosterone Cypionate	253.65 mg/mL	Chief Lab	Yes	Testosterone Cypionate	No - overdosed	434 mg/mL (± 5% error = 412–456)
Trenbolone Enanthate	200 mg/mL	OZPharm Labs	Yes	Trenbolone Enanthate	Yes	199 mg/mL (± 5% error = 189–209)
Oxandrolone (Anavar)	10 mg	Swiss Pharm	No	Stanozolol	No - underdosed	3.3 mg
*Oxymetholone (Anadrol)	20 mg	Eagle1 Laboratories	-	-	N/A	-
Methenolone Enanthate (Primobolan)	200 mg/mL	Not Provided	Yes	Methenolone Enanthate	N/A	-
Oxandrolone (Anavar)	10 mg	Not Provided	No	Oxandrolone, Testosterone	No - underdosed	7 mg Oxandrolone, 1.6 mg Testosterone
Mesterolone (Proviron)	25 mg	Viropace	Yes	Mesterolone	No - underdosed	22 mg
Testosterone Enanthate	400 mg/mL	Eagle1 Laboratories	Yes	Testosterone Enanthate	Yes	411 (± 5% error = 390–432)
Nandrolone Decanoate (Deca)	200 mg/mL	Eagle1 Laboratories	Yes	Nandrolone Decanoate	N/A	-
Drostanolone Enanthate (Masteron)	200 mg/mL	Eagle1 Laboratories	Yes	Drostanolone Enanthate	No - overdosed	215 mg/mL (± 5% error = 204–226)
Testosterone Enanthate	250 mg/mL	Eagle1 Laboratories	Yes	Testosterone Enanthate	Yes	238 mg/mL (± 5% error = 226–250)
Nandrolone Phenylpropionate (NPP)	100 mg/mL	HYBRID	Yes	Nandrolone Phenylpropionate	No - underdosed	93 mg/mL (± 5% error = 88–98)
Nandrolone Phenylpropionate (NPP)	100 mg/mL	HYBRID	Yes	Nandrolone Phenylpropionate	No - overdosed	116 mg/mL (± 5% error = 110–122)
Drostanolone Enanthate (Masteron)	200 mg/mL	HYBRID	Yes	Drostanolone Enanthate	Yes	201 mg/mL (± 5% error = 191–211)
Trenbolone Enanthate	200 mg/mL	ShelbyPharma Corp	Yes	Trenbolone Enanthate	Yes	204 mg/mL (± 5% error = 194–214)
Testosterone Enanthate	250 mg/mL	ShelbyPharma Corp	Yes	Testosterone Enanthate	No - underdosed	229 mg/mL (± 5% error = 216–240)
Drostanolone Enanthate (Masteron)	200 mg/mL	ShelbyPharma Corp	Yes	Drostanolone Enanthate	Yes	203 mg/mL (± 5% error = 193–213)
Testosterone Enanthate	250 mg/mL	Australian Genetic Pharmaceuticals	No	Testosterone Cypionate	No - different PIED	253 mg/mL (± 5% error = 240–267)
Trenbolone Enanthate	200 mg/mL	Platinum	Yes	Trenbolone Enanthate	No - underdosed	167 mg/mL (± 5% error = 159–175)
Trenbolone Enanthate	200 mg/mL	Sparta	Yes	Trenbolone Enanthate	Yes	200 mg/mL (± 5% error = 190–210)
**Wave 2: 14 JUNE 2024–16 AUGUST 2024**						
Sustanon 250	250 mg/mL	Molecule	No	Testosterone Enanthate	No - different PIED	62 mg/mL (± 5% error = 59–65)
Trenbolone Enanthate	200 mg/mL	Anabolic Research Lab	Yes	Trenbolone Enanthate	No - underdosed	34 mg/mL (± 5% error = 32–36)
Tamoxifen (Nolvadex)	20 mg	Alphapharm	Yes	Tamoxifen	YES	20 mg
Exemestane (Aromasin)	25 mg	Platinum Anabolics	Yes	Exemestane	No - underdosed	15.5 mg
Methandienone (Dianabol)	Not Provided	Keifei Pharma	Yes	Methandienone	N/A	8.6 mg
Methandienone (Dianabol)	10 mg	Keifei Pharma	No	Oxymetholone, Methandienone	No - different PIED	< 0.1 mg Methandienone No Reference materials for oxymetholone.
Oxymetholone (Anadrol)	50 mg	Not Provided	No	Methandienone, Oxymetholone	No - different PIED	2.5 mg Methandienone. No Reference materials for oxymetholone.
*RAD140 (Testolone)	Not Provided	-	-	-	-	Carrier = PEG type compound
Trenbolone Acetate	100 mg/mL	Balkan Pharmaceuticals	Yes	Trenbolone Acetate	No - underdosed	45 mg/mL (± 5% error = 43–47)
Nandrolone Decanoate (Deca)	300 mg/mL	Shelby Pharma Corp	No	Nandrolone Decanoate	N/A	-
*Clenbuterol	125 µg/mL	Azelique	-	-	-	Carrier = PEG type compound
Sustanon 300 mg/ml (TestPP 70 mg/mL, Test Isoc 70 mg/mL, Test Prop 60 mg/mL, Test Deca 100 mg/mL)		King 300 Labs	No	Testosterone Cypionate	No - different PIED	165 mg/mL (± 5% error = 157–173)
Testosterone Enanthate	250 mg/mL	Not Provided	Yes	Testosterone Enanthate	No - underdosed	160 mg/mL (± 5% error = 152–168)
Testosterone Enanthate	250 mg/mL	Green Dispensary Pharmacy	Yes	Testosterone Enanthate	Yes	249 mg/mL (± 5% error = 237–261)
Anastrozole (Arimidex)	2 mg	Not Provided	Yes	Anastrozole	N/A	-
MK-677 (Ibutamoren)	Not Provided	Not Provided	Yes	MK677	N/A	2 mg
MK2866 (Ostarine) and MK677 (Ibutamoren)	Not Provided	Not Provided	Yes	MK2866, MK677	N/A	5.1 mg MK2688 and 0.1 mg MK677
Methenolone Enanthate (Primobolan)	100 mg/mL	Chief Labs	Yes	Methenolone Enanthate	N/A	-
Drostanolone Enanthate (Masteron)	200 mg/mL	Polar	Yes	Drostanolone Enanthate	No - overdosed	257 mg/mL (± 5% error = 244–270)
Methenolone Enanthate (Primobolan)	200 mg/mL	Polar	Yes	Methenolone Enanthate	N/A	-
Tamoxifen (Nolvadex) [Raw powder]	Pure	Not Provided	Yes	Tamoxifen	Yes	99% purity
Oxandrolone (Anavar) [Raw powder]	Pure	Not Provided	Yes	Oxandrolone	Yes	97% purity
Exemestane (Aromasin) [Raw powder]	Pure	Not Provided	Yes	Exemestane	Yes	95% purity
Unk. Peptide [CHEQ Result = LGD 4033 (Ligandrol)] [Raw powder]	Pure	Not Provided	Yes	LGD4033	Yes	99% purity
Unk. Peptide [CHEQ Result = RAD140 (Testolone)] [Raw powder]	Pure	Not Provided	Yes	RAD140	No - underdosed	77% purity

Note. *Unable to analyse owing to limitation in resources and equipment

### Phase 2: follow-up interviews

#### Participant recruitment

Following the dissemination of results, participants were recruited for qualitative interviews to explore how the findings influenced their perceptions and behaviours related to AAS use. Eligibility criteria included prior engagement with Wave 1, either through submitting samples or accessing the published test results. Recruitment was conducted through community networks, including The Loop Australia, the Queensland Injectors Health Network, and Queensland Injectors Voice for Advocacy and Action.

#### Data collection

Semi-structured interviews were conducted by an author (SR) via Microsoft Teams, with all session’s audio recorded with participant consent and transcribed for analysis. Interview questions were collaboratively developed with AAS community members to ensure relevance. In both the original and follow up interviews, participants were asked about their perceived susceptibility to health risks associated with AAS use: *Do you believe you are at risk of experiencing negative health effects from using AAS? Why or why not?* To assess whether recent test results influenced risk perception, they were also asked: *When thinking about the Wave 2 results*,* has your perception of your risk to experiencing negative health effects changed? If so*,* how?* Additionally, impact of the pilot and its’ expansion were explored through questions such as: *Did the recent results regarding AAS in circulation prompt you to reconsider your usage of AAS? If yes*,* in what way? If no*,* why not?* Finally, participants were asked to compare their responses to previous findings: *How did the Wave 2 results differ from the Wave 1 results when considering your usage of AAS?* These questions aimed to examine how evolving data on AAS product variability influenced risk perception and decision-making.

#### Data analysis

SPSS (v30, IBM) was used to analyse the demographic information submitted at time of sample drop off. Interview data were analysed using NVivo 12 (QSR), applying a mixed deductive-inductive approach under the Health Belief Model (HBM) framework [28]. Initially, TP used a deductive coding phase categorised responses according to key HBM constructs, including perceived susceptibility, severity, benefits, and barriers associated with AAS use and testing outcomes. This was followed by TP employing an inductive, line-by-line analysis using iterative categorisation [29] to refine and expand thematic insights. To enhance analytical depth, positionality reflections were incorporated throughout. SR, a PhD student without AAS experience, and TP, a peer researcher with lived-living AAS expertise, provided complementary perspectives to contextualise findings. The lead author (TP) employed abductive conceptualisation [30], drawing from participant narratives, theoretical insights, and prior findings to refine identified and developed themes. The lead author (TP) employed abductive conceptualisation (Neale, 2021), drawing from participant narratives, theoretical insights, and prior findings to refine identified and developed themes.

This process was further supported by critical discussions within the research team to ensure rigour, challenge biases, and capture the nuanced complexities of AAS use. The research team combined peer, academic, and technical expertise. Four authors (TP, EK, BW, MB) are peer researchers, with TP bringing lived-living experience of AAS use. RC, CF, and GD offered expertise in steroid research and harm reduction service delivery. SC, AW, MH, and KB contributed chemical analysis expertise, while CP, JF, SR brought drug and alcohol research experience and allyship to peer-led approaches. Findings are presented below in five overarching thematic categories.

## Results

### Sample composition

Participants submitted 58 AAS samples to CheQpoint between 19th April–16th August 2024. In this group, 37 samples were tried prior to submission, and two of these samples were reported to induce disappointing or different effects. In terms of reasons for sample submission, 46 samples were submitted owing to general interest or curiosity, four samples were submitted as participants wanted information or advice, 3 samples were reported as unexpected compounds, and 5 samples were submitted without a reported reason.

In response to community feedback, data from a total of 58 samples submitted during Waves 1 and 2 were presented to the community. Of these, 46 were analysed qualitatively for matched compounds, and 36 quantitatively for concentration. While participants submitted a variety of substances, only AAS were within the scope of validated testing methods at the time of analysis. Nine samples of human growth hormone were not analysable as the research team had not developed appropriate methods. Of the 46 analysable samples, 9 exhibited presence issues, while 15 demonstrated purity issues. Of the 15 oral products tested, 10 contained the expected compound, while 5 deviated from the expected compounds. For 31 injectable products, 28 matched the expected AAS, though 11 were either underdosed or overdosed. All 5 raw AAS powders contained the expected compound, with one found underdosed.

### Follow-up interviews

Forty semi-structured interviews were conducted with 25 participants (*Mean*_age_ = 35.9 years; 20 male, 5 female). Of these, 15 participants (*Mean*_age_ = 36.3; 11 male; 4 female) completed follow-up interviews with a median length of 58 min (range = 34–70 min), and a 60% retention rate (see, Table 2). Participants reported current use of AAS at interview. The most frequent number of AAS compounds used was one (*n* = 5) or two (*n* = 4). Compounds were acquired through illicit avenues, such as community networks (*n* = 7), and online markets (*n* = 2), or a combination of illicit avenues and licit prescriptions (*n* = 4).

**Table 2:** Participant summary at wave 1 and wave 2

		Wave 1	Wave 2
Pseudonym	Gender	Age	Age
Victor	Male	29	29
Caleb	Male	42	42
Oliver	Male	37	37
Ava	Female	44	44
Albert	Male	40	40
Alexander	Male	31	31
Rose	Female	44	44
Asher	Male	27	28
Douglas	Male	28	29
Damien	Male	37	38
Luci	Female	45	45
Penny	Female	33	34
Brock	Male	34	35
Zane	Male	31	31
Ben	Male	40	40
Royce	Male	49	-
Harry	Male	22	-
Ethan	Male	28	-
Hugh	Male	41	-
Dante	Male	39	-
Samson	Male	39	-
Leo	Male	32	-
Rex	Male	41	-
Felix	Male	32	-
Grace	Female	32	-

#### ‘Enhanced’ testing results and the perceived risks

Participants reflected on how Wave 2 testing results intersected with their pre-existing risk perceptions, particularly in relation to dosage accuracy and product consistency. For many, the results reinforced concerns about under-dosing, with many substances showing only fractional inconsistencies. However, others noted that some deviations were far more extreme, creating a greater potential for harm.

Ava [44, female]: I’m looking at them [wave 2 results] […] they’re under dosed [containing less than the labelled purity], which is a lot of them, it was a fractional underdosing. There were also others there and some are wildly different.

In addition, participants noted that the patterns of misrepresentation in Wave 2 were not entirely surprising, particularly for certain substances that have a reputation for inconsistency.

Damien [37, male]: I’m not surprised in terms of the particular substances. I’m not surprised [of] that Sustanon (Testosterone blend) in Wave 2.

While testing services provide empirical confirmation, some participants had already anticipated issues with specific compounds, indicating that knowledge of risk is shaped by both experience and peer networks. Despite this, under-dosing remained a concern, particularly for compounds that require precise dosing to achieve the intended effects. The potential for receiving less effective product was not only an economic issue but also a performance-related risk, as participants relied on expected pharmacological effects to guide their usage.

Albert [40, male]: I wouldn’t want my tren [trenbolone] underdosed if I wanted to use it. I would use it at a very miniscule dose anyway. But if I wanted to use it, I still want it to have the full effect.

Beyond concerns around dosage inconsistencies, some participants articulated how incorrect compound identification could have significant physiological consequences such as gynecomastia (breast tissue growth in men). For those engaged in structured performance enhancement, such as competitive bodybuilding or powerlifting, the substitution of one compound for another could drastically alter their hormonal balance, with serious side effects.

Caleb [42, male]: An example would be a competition bodybuilder or powerlifter in a prep [competition preparation phase] they might run testosterone, Masteron [drostanolone], NPP [nandrolone phenyl-propionate], and Tren [trenbolone], and Anadrol [oxymetholone] all within like a two-week period. Say that masteron turns out to be Deca [nandrolone] or the NPP turns out to be test [testosterone]. That could be the difference of them getting gyno [gynocomastia] or not or coming out with severe acne because they could potentially be tripling their test.

This reinforces how AAS consumers engage in careful dosing science, using specific combinations of compounds to regulate their physiological responses and optimize outcomes while managing potential harms. Testing services, therefore, play a critical role in preventing unintended and potentially harmful hormonal fluctuations. This ‘science’ of dosing is crucial for athletes who are not seeking just a high, but rather a controlled, strategic approach to enhance performance and minimise risks.

#### Testing as a tool for informed decision-making

Participants were impressed with the improvement in accurate testing data and articulated how this played a crucial role in shaping decision-making around AAS use. For many participants, the ability to verify product composition is a key benefit, offering reassurance in an environment often rife with misinformation.

Caleb [42, male]: I think the work that’s being done is great. There’s so much misinformation out there that when you get a chance to get the data like that presented to you, it’s just a great tool to go through and show people what they’re taking.

Another key benefit that emerged was the reassurance that testing provides about the legitimacy of substances being circulated. Participants recognised that while the unregulated nature of AAS markets presents risks, our improved testing results offered a level of oversight, helping to ensure that not all products are fraudulent or harmful.

Ben [40, male]: It’s nice to see that there’s some efficacy on the streets and people aren’t just taking advantage.

By confirming that a portion of products contain what they claim to, testing services provide consumers with an additional layer of confidence when sourcing substances. Beyond reassurance, participants also described how access to accurate testing data could shape their decision-making regarding health risks. Testing data prompted consumers to reassess their level of risk exposure, demonstrating that having information leads to a deeper reflection on potential harms.

Douglas [28, male]: Seeing this, I would be more inclined to test them [steroids] so I know exactly what I’m getting. It makes me think about the health risks a little bit more than what they already are because I’m already taking a risk. And then if what’s in them isn’t even the right thing, like I might not even be preparing for the right thing.

This demonstrates how harm reduction services can actively shape people’s risk management practices.

#### Barriers to acting on testing results in an unregulated market

Despite these benefits, some participants also identified barriers that complicate the ability to fully act on testing results. One such barrier is the lack of consumer protections in the illicit AAS market.

Alexander [31, Male]: It is what it is. I’m very accepting of the fact that this isn’t Kmart and I can’t go back and return something if it’s faulty. I’m totally OK with it being as is because the perceived benefits to me outweigh any potential risk.

While testing can confirm a problem, it does not necessarily provide a straightforward solution, underscoring the practical limitations of what consumers can do with test results. Another major barrier identified by some participants was the turnaround time for testing results. Several people expressed concerns that testing services may not always align with the realities of AAS purchasing and usage strategies.

Ava [44 Female]: It would be nice to know quickly what you have because you’re not always going to buy something months before you go to start using it […] not necessarily like myself where you’re going to arrange what you need for the next, you know, 12 months of competition. I might need to start my [AAS] cycle before I get that information back.

Some participants articulated the complex decision-making process that follows an unexpected test result. If a product is misrepresented, consumers face difficult choices: adjust their dosage based on assumptions, discard their product and repurchase, and/or wait additional time for new testing, with each option presenting its own barriers, whether financial, logistical, or related to training schedules.

Ava [44 Female]: Then if you get information that contradicts what you believe you have, then that’s a fork in the road decision of, well do you adjust the dosage to make sure that you’re getting the dosage that you believe that you’ve got? Do you bin what you have and buy new product and hope that it’s better? And then maybe wait another three months to get information? I think that lag time certainly isn’t beneficial necessarily to the person.

This demonstrates how structural delays in testing services can complicate consumers’ ability to act on harm reduction strategies effectively.

#### Underground ‘branding’ as cues to action in risk management

Wave 2 testing results introduced reporting of underground product branding. This addition provided AAS consumers with an extra layer of information, allowing them to make assessments regarding the reliability of specific brands. For some participants, branding reports served as a direct cue to action, influencing their purchasing decisions by helping them identify which brands were more likely to provide accurate products.

Oliver [37, male]: I can see the brands and it’s like [I’m] probably going to stay away from that brand now. I see some other brands that are good. Concentration match, yes. I’m getting what I’m buying. I’m going to steer more towards the ones that have come back good.

Beyond individual decision-making, participants also recognised the potential for branding reports to contribute to broader, community-level knowledge. By tracking brand trends over time, consumers could collectively build a more accurate picture of product reliability within the AAS market.

Brock [34, male]: Well [it] helps from a macro level. If we have enough substances we can start making more informed decisions. But we [also] need results on this specific bottle, if you can tell me what’s in that one, I could make the best decision.

This highlights a key tension: while branding reports provide a useful overview, they do not eliminate the uncertainty associated with individual batches. Participants acknowledged that while broad trends were helpful, batch-specific testing remained essential for precise decision-making. This concern was reinforced by participants who had seen firsthand how brand reputation did not always equate to consistency. Even when a brand had produced high-quality products in the past, there was no guarantee that all its products would meet the same standard.

Rose [44, female]: For me it confirms that you can’t tell, you just don’t know. Like the Keifei pharma ones. Two Dianabol [methandrostenedione] ones, right. And one’s not [Dianabol]. Even when a brand does have good stuff their other stuff can be crap. So buy from someone who I know as a supplier and regularly tests everything […] check every single batch.

The presence of branding reports provides some guidance, but ultimately, some consumers relied on personal networks and peer-led quality control measures to mitigate risk further. This reflects an intersection between formalised testing and informal community verification systems, reinforcing the role of social trust in harm reduction strategies. Participants also reflected on how Wave 2 results reinforced the need for ongoing vigilance and due diligence.

Luci [45, female]: On the data findings from between wave one and wave two […] I would have expected more to be swapped out for other things. The Wave 2 results just reinforce the need to have that due diligence and to have your own harm minimisation and risk mitigation strategies in place.

This demonstrates how testing acts as a cue to action—not just in immediate decision-making but in reinforcing long-term strategies for risk management. These cues interact with perceived susceptibility and severity, as consumers weigh the risks of product variability and the potential health consequences of using misrepresented substances.

#### Self-efficacy in navigating use and health

A key determinant of harm reduction effectiveness is not just access to information but the ability to apply that knowledge in meaningful ways. While testing results provide valuable data, their impact depends on the consumer’s capacity to interpret and integrate them into their broader practices.

Alexander [31, Male]: I think information is great but understanding the application of that knowledge is where the power will come from. So, we can individually sample all these, but if the person doesn’t know what to do with that information there is still the same problem.

This highlighted a critical limitation in steroid testing: providing data without corresponding education or practical guidance may not translate into safer practices. One of the most concrete examples of this challenge relates to dosage miscalculations due to inconsistent product concentrations. The ability to accurately adjust related medications, such as aromatase inhibitors used to manage estrogenic side effects, is contingent on understanding how underdosing or overdosing impacts hormone balance.

Oliver [37, male]: Let’s say that you’ve got an underdosed [product] and you think you’re taking 250 mg/ml? It’s actually 150 mg/ml. So, you’re taking the amount of aromatase inhibitors you think you should be taking for that amount of dosage […] and then you’re going to not feel very good. The only way to check all those things is if we do bloods.

Recognising that AAS dosages may not align with expectations requires proactive adjustments, and without regular blood testing, consumers may not realise the extent of miscalculations until they experience adverse effects. Another dimension of self-efficacy involved recognising physical cues and linking them back to dosing inconsistencies. Some participants described how users often misattribute physiological changes to a specific compound, without realising that inconsistencies in concentration may be altering their actual intake.

Caleb [42, male]: It might be they thought that 140 milligrams of Anavar [oxandrolone] a week was going to dry them out, but they ended up taking 40 milligrams of Winstrol [stanzolol] and it made them look bloated. That might change their protocol for the next time without even realising going “I didn’t respond to Anavar”. And it’s like, “OK, so we’ll cross that off the list” and then they go to take Winstrol again at 10 milligrams and it actually comes in at 10. And previously they’ve taken it 3.3 and end up taking three times the dosage straight away […] serious follow on health effects.

Developing self-efficacy in monitoring requires not only access to testing but also consistent tracking of responses and understanding how different compounds interact with the body. Finally, participants acknowledged that responsible AAS use involves a comprehensive set of self-monitoring behaviours beyond testing.

Luci [45, female]: If you’re going to go down this path of anabolics use there’s an assumed responsibility that you’re going to organise to have your blood work done, to not be continuously cycling, to have breaks, to make sure that if you’re a male doing your post cycle therapy, making sure that you’re doing cardio and that you’re drinking enough water and taking additional supplements.

Even when individuals perceive risks (e.g., inconsistencies in dosing, potential health harms), their ability to take effective action depends on their belief in their capacity to implement harm reduction strategies successfully. Testing and information access are crucial, but their impact is maximised when individuals possess the skills and confidence to translate knowledge into practical action.

## Discussion

This follow-up study reflects that there are significant discrepancies in the composition and concentration of AAS circulating in the market, as demonstrated through both chemical testing and consumer interviews. Our results indicate that while a majority of products, especially raw AAS powders, contained the expected compounds, there were notable variations in the concentration of injectable and oral formulations. These discrepancies are less than those reported for UK markets [4], and included instances of both underdosing and overdosing, the latter being a novel finding. These findings underscore persistent quality control issues in the unregulated AAS market globally [5] and continue to raise concerns about the potential health risks associated with using unregulated AAS.

Participants’ reflections indicate that the testing results, particularly from Wave 2, reinforced pre-existing concerns regarding product reliability, dosage accuracy, and the potential for misrepresented substances. This heightened awareness appears to have influenced individual risk assessments and has, in some cases, prompted consumers to reconsider their dosing strategies and purchasing behaviours. The integration of branding reports in the testing process particularly emerged as an important cue to action, with many participants indicating that they now use brand information as an additional layer in their decision-making process. Importantly, these findings highlight the broader benefits of AAS testing beyond individual results. By analysing samples across multiple Waves, this approach provides a market-level overview of AAS trends within specific regions, offering consumers valuable insights into product quality, purity, and branding reliability. This collective knowledge strengthens harm reduction efforts, allowing consumers to anticipate risks and make more informed decisions. The incorporation of branding reports into testing protocols represents a novel advancement, enhancing consumer agency and fostering a more proactive approach to harm reduction.

While many participants valued the enhanced accuracy of the testing results and indicated that they used this information to adjust their AAS usage, some expressed frustration over systemic barriers, such as lengthy turnaround times for test results, which is one of the technological limitations of current AAS offsite testing approaches [4]. These constraints underscore the urgent need for improved testing infrastructure, including faster, more accessible methodologies that better align with consumer needs. Strengthening testing efficiency will enhance the utility of AAS analysis, supporting more timely and actionable harm reduction strategies. Furthermore, the study confirms the pivotal role of community networks in shaping perceptions of risk, quality, and harm reduction strategies [22, 31–33]. In a political environment where AAS remain highly criminalised in Australia [19] and stigma is pervasive [34], peer networks serve as critical infrastructures for navigating an unregulated market. Participants frequently relied on a combination of formal testing data and peer-reported information, illustrating the co-production of harm reduction knowledge through lived-living experience. This community-led approach not only enhances access to real-time, contextually relevant information but also mitigates the gaps left by institutional responses. Strengthening peer-driven harm reduction initiatives, particularly in contexts where criminalisation limits formal public health promotion and engagement, emphasises the necessity of embedding lived-living expertise in AAS harm reduction efforts.

### Implications and future directions

These findings underscore the urgent need for more accessible, community-integrated AAS testing services in Australia. The enhanced testing methodologies introduced in Wave 2 demonstrate the potential for refining harm reduction approaches by offering consumers more reliable, actionable information. Beyond individual testing, the incorporation of branding reports suggests a novel opportunity for community-wide harm reduction. By mapping trends in AAS markets within specific geographic regions, these insights enable consumers to make informed choices that extend beyond isolated test results. However, the next step must be to transition from batch testing to individualised drug checking, aligning AAS testing with existing models used in broader drug checking programs [14, 35]. This shift would ensure equitable healthcare delivery, allowing consumers to receive direct, personalised feedback on their substances—critical for informed decision-making and harm reduction.

Beyond AAS, expanding testing programs to include other enhancement drugs (e.g., human growth hormone) is essential. These substances are widely used yet remain understudied [36], leaving consumers with little to no quality control mechanisms. A broader testing program would provide a comprehensive picture of the unregulated market, addressing gaps in both research and service provision. Additionally, the aggregation and community-level dissemination of testing data offers a novel harm reduction intervention in itself. By sharing trends in purity, adulteration, and branding across geographic regions, such reporting fosters collective risk reduction—where consumers actively discuss, interpret, and apply the findings to their own use. This kind of community-driven surveillance has proven effective in other drug markets [37, 38], yet Australia lacks a dedicated AAS monitoring system. Implementing a structured, ongoing reporting mechanism would not only inform consumers but also shape harm reduction policy by highlighting shifts in product composition and emerging risks.

### Limitations

Notably, our testing approach evolved considerably from Wave 1 to Wave 2. In Wave 1, purity analysis was limited, with only one sample undergoing such assessment, whereas in Wave 2, we re-analysed all Wave 1 samples alongside newly collected ones and expanded our testing protocol to include a comprehensive purity analysis. This enhanced the depth of our data and also allowed the research team to include previously untested underground brands, thereby broadening our understanding of product variability in unregulated supply channels. However, technological and logistical barriers, such as lengthy turnaround times, highlight the necessity of investing in testing infrastructure that is both responsive and tailored to the needs of people who use AAS.

### Conclusions

This study demonstrates that chemical analysis and reporting can be a powerful tool in AAS harm reduction. Incorporating branding reports and expanded substance testing yielded valuable, in-depth follow-up feedback that improved accuracy and reinforced consumers’ awareness of product variability and risk. Moreover, the engagement of consumers with our services was influenced by the availability and accessibility of such data, highlighting the potential for drug checking services to facilitate greater consumer health engagement. Consumers indicated that testing the AAS products affected their use. Expanding drug checking services to include AAS and establishing a structured monitoring system could further enhance harm reduction efforts.

## Electronic supplementary material

Below is the link to the electronic supplementary material.

Supplementary Material 1

## Data Availability

Data regarding chemical analysis results is publicly available: https://hi-ground.org/app/uploads/2024/10/ROIDCheck-Wave-2-Results.pdf. The interview data is available from corresponding author on reasonable request.
