# Supplementary material for: Anabolic-androgenic steroid testing as a tool for consumer engagement and harm reduction: a sequential explanatory mixed-method study
Source: Harm Reduct J. 2025 Jul 4;22:114. doi: 10.1186/s12954-025-01270-4 (PMC12231991; doi:10.1186/s12954-025-01270-4)
Supplement: Supplementary file 1 — Supplementary Material 1 [file 12954_2025_1270_MOESM1_ESM.docx]

**Appendix A.**

### COREQ Criteria Checklist

**Developed from:**

Tong A, Sainsbury P, Craig J. Consolidated criteria for reporting qualitative research (COREQ): a 32-item checklist for interviews and focus groups. *International Journal for Quality in Health Care*. 2007. Volume 19, Number 6: pp. 349 – 357

| **No.** | **Item** | **Guide Questions/Description** | **Notes and/or Section Reported in** |
| --- | --- | --- | --- |
| **Domain 1: Research team and reﬂexivity** | | | |
| *Personal Characteristics* | | | |
| 1 | Interviewer/  facilitator | Which author/s conducted the interview or focus group? | Steph Reeve with guidance from Timothy Piatkowski |
| 2 | Credentials | What were the researcher’s credentials? E.g. PhD, MD | Timothy Piatkowski has his PhD and Steph Reeve is a PhD student. |
| 3 | Occupation | What was their occupation at the time of the study? | The interviewer is a PhD student. Timothy Piatkowski is a full-time academic. |
| 4 | Gender | Was the researcher male or female? | Timothy Piatkowski is a male and Steph Reeve is a female. |
| 5 | Experience and training | What experience or training did the researcher have? | The interviewer has experience in conducting research with people who use illicit drugs and has worked closely with Timothy Piatkowski on studies related to people who use drugs. |
| *Relationship with participants* | | | |
| 6 | Relationship established | Was a relationship established prior to study commencement? | Not for Steph Reeve. Initial contact was only to establish interest in the study by providing the Participant Information Sheet and to organise a time for interview.  Timothy Piatkowski has a longstanding personal and professional connection and network within steroid-using communities. |
| 7 | Participant knowledge of the interviewer | What did the participants know about the researcher? e.g. personal goals, reasons for doing the research | Only information provided in the Participant Information Sheet and Interview Guide. See Method section. |
| 8 | Interviewer characteristics | What characteristics were reported about the interviewer/facilitator? e.g. Bias, assumptions, reasons and interests in the research topic | Timothy Piatkowski and Steph Reeve discuss positionality regarding the work. See Method – Data Analysis section. |
| **Domain 2: Study design** | | | |
| *Theoretical framework* | | | |
| 9 | Methodological orientation, ontological or epistemological basis | What methodological orientation was stated to underpin the study? e.g. grounded theory, discourse analysis, ethnography, phenomenology, content analysis | Extended-pilot study, exploratory in nature. See Method section for more information. |
| *Participant selection* | | | |
| 10 | Sampling | How were participants selected? e.g. purposive, convenience, consecutive, snowball | Purposive and snowball sampling. See Method section for more information. |
| 11 | Method of approach | How were participants approached? e.g. face-to-face, telephone, mail, email | Via email. Described in the Method section. |
| 12 | Sample size | How many participants were in the study? | A total of 25 people agreed to participate in the qualitative element of this study. |
| 13 | Non-participation | How many people refused to participate or dropped out? Reasons? | 40 interviews were conducted, 25 interviews and 15 follow-up interviews. See Methods section for more information. |
| 14 | Setting of data collection | Where was the data collected? e.g. home, clinic, workplace | Collection of data was via the recording of a Microsoft Teams interview with participants. The researcher recommended participants to be a in quiet place free from distractions during the duration of the interview. |
| 15 | Presence of non-participants | Was anyone else present besides the participants and researchers? | Unable to be determined as interviews were conducted over Teams. No other persons were present on the researcher’s end. |
| 16 | Description of sample | What are the important characteristics of the sample? e.g. demographic data, date | Refer to Results section. |
| *Data collection* | | | |
| 17 | Interview guide | Were questions, prompts, guides provided by the authors? Was it pilot tested? | Refer to Methods. The Interview Guide was piloted through engagement with community and iterative discussions between Dr Piatkowski and consumers in his personal networks. See Method section for more information. |
| 18 | Repeat interviews | Were repeat interviews carried out? If yes, how many? | No repeat interviews were conducted with participants. |
| 19 | Audio/visual recording | Did the research use audio or visual recording to collect the data? | Video and audio recording. See Method section for more information. |
| 20 | Field notes | Were ﬁeld notes made during and/or after the interview or focus group? | Field notes were taken during and after the interviews. See Method section for more information. |
| 21 | Duration | What was the duration of the interviews or focus group? | Median 58 minutes. |
| 22 | Data saturation | Was data saturation discussed? | Yes. See Method section. |
| 23 | Transcripts returned | Were transcripts returned to participants for comment and/or correction? | Transcripts were offered to participants for review. No participants took up the offer. |
| **Domain 3: Analysis and findings** | | | |
| *Data analysis* | | | |
| 24 | Number of data coders | How many data coders coded the data? | One data coder coded the data in collaboration with the whole research team. |
| 25 | Description of the coding tree | Did authors provide a description of the coding tree? | Yes. See Method section. |
| 26 | Derivation of themes | Were themes identiﬁed in advance or derived from the data? | Theme-categories were derived from the data. See Method section on the process. |
| 27 | Software | What software, if applicable, was used to manage the data? | Qualitative data analysis software NVivo Version 12 was used to organise the interview transcripts. |
| 28 | Participant checking | Did participants provide feedback on the ﬁndings? | No, but preliminary findings were provided to participants. |
| *Reporting* | | | |
| 29 | Quotations presented | Were participant quotations presented to illustrate the themes/ﬁndings? Was each quotation identiﬁed? e.g. participant number | Yes - pseudonyms. See Results section. |
| 30 | Data and ﬁndings consistent | Was there consistency between the data presented and the ﬁndings? | Yes, findings were directly derived from the data. See Results and Discussion sections for more information. |
| 31 | Clarity of major themes | Were major themes clearly presented in the ﬁndings? | Yes. Categories were discussed in the Results section. See Results section for more information. |
| 32 | Clarity of minor themes | Is there a description of diverse cases or discussion of minor themes? | Data was highly homogenous. Minor discussion of diverse cases occurs in the Results section. |
